# Mitochondrial DNA variation and phylogeography of native Mongolian goats

**DOI:** 10.5713/ajas.19.0396

**Published:** 2019-10-21

**Authors:** Onolragchaa Ganbold, Seung-Hwan Lee, Woon Kee Paek, Munkhbaatar Munkhbayar, Dongwon Seo, Prabuddha Manjula, Tamir Khujuu, Erdenetushig Purevee, Jun Heon Lee

**Affiliations:** 1 Laboratory of Animal Molecular Genetics, Division of Animal & Dairy Science, Chungnam National University, Daejeon 34134, Korea; 2 Department of Biology, Mongolian National University of Education, Ulaanbaatar 210685, Mongolia; 3 Daegu National Science Museum of Korea, Daegu 43014, Korea

**Keywords:** Genetic Diversity, Mitochondrial DNA, Native Goats, Phylogenetic Relationships, The Origin

## Abstract

**Objective:**

Mongolia is one of a few countries that supports over 25 million goats, but genetic diversity, demographic history, and the origin of goat populations in Mongolia have not been well studied. This study was conducted to assess the genetic diversity, phylogenetic status and population structure of Mongolian native goats, as well as to discuss their origin together with other foreign breeds from different countries using hypervariable region 1 (HV1) in mtDNA.

**Methods:**

In this study, we examined the genetic diversity and phylogenetic status of Mongolian native goat populations using a 452 base-pair long fragment of HVI of mitochondrial DNA from 174 individuals representing 12 populations. In addition, 329 previously published reference sequences from different regions were included in our phylogenetic analyses.

**Results:**

Investigated native Mongolian goats displayed relatively high genetic diversities. After sequencing, we found a total of 109 polymorphic sites that defined 137 haplotypes among investigated populations. Of these, haplotype and nucleotide diversities of Mongolian goats were calculated as 0.997±0.001 and 0.0283±0.002, respectively. These haplotypes clearly clustered into four haplogroups (A, B, C, and D), with the predominance of haplogroup A (90.8%). Estimates of pairwise differences (*Fst*) and the analysis of molecular variance values among goat populations in Mongolia showed low genetic differentiation and weak geographical structure. In addition, Kazakh, Chinese (from Huanghuai and Leizhou), and Arabian (Turkish and Baladi breeds) goats had smaller genetic differentiation compared to Mongolian goats.

**Conclusion:**

In summary, we report novel information regarding genetic diversity, population structure, and origin of Mongolian goats. The findings obtained from this study reveal that abundant haplogroups (A to D) occur in goat populations in Mongolia, with high levels of haplotype and nucleotide diversity.

## INTRODUCTION

The domestic goat (*Capra hircus*) is one of the earliest domesticated animals. Archaeological evidence suggests that the Fertile Crescent region in the Near East (NE) was the domestication center of domestic goats, and that this event occurred more than 10,000 years ago [1,2]. Meanwhile, some studies suggest a second, independent domestication event for Cashmere-like goat breeds in Pakistan [3]. Recently, goat breeds are recognized as one of the most important (source of meat, milk, and fiber) and adaptable (adapted to savannas, desert, high mountain, and cold winters) domestic animals in the world, but particularly in countries of Africa and Asia. Three wild species of *Capra*, bezoar (*C. aegagrus*), ibex (*C. ibex*) and markhor (*C. falconeri*) are phylogenetically close to the domestic type (*C. hircus*), and it has been suggested that all these wild species contributed to the genetic composition of domestic goats [4–6]. Among these candidates, *C. aegagrus* is widely agreed to be the wild ancestor of domestic goats, as all haplogroups of domestic goat occur within current *C. aegagrus* populations [6,7].

Mitochondrial DNA (mtDNA) is a critical tool for tracing evolutionary and population genetics of most domesticated animals. Also, mtDNA diversity is vital in assessing the maternal origin, phylogeny, and population structure of all domestic animals [8–13]. Previous studies of goat mtDNA (control region) revealed a total of six haplogroups (also known as lineages; A, B, and C by Luikart et al [14]; D by Sultana et al [15]; F by Sardina et al [16]; and G by Naderi et al [6]). Of these, haplogroup A is the most widely distributed through the world (approximately 90% of total goats belong to A haplogroup), while rest of haplogroups (approximately 10% of total goats) have limited distribution areas with B haplogroups occurs only in southern and eastern Asia [17]. These previous studies [6,14–16] also revealed their relatively weak phylogeographic structure across the world.

Due to their high economic importance, goats are considered as an important livestock species in Mongolia. In 2017, the estimated population of goats in Mongolia was 27.3 million, 41.3% of the total of 66.7 million heads of livestock [18]. All goats in Mongolia are reared as a source of cashmere in the country, while they are used relatively less as a source of milk and meat. For this reason, Mongolia is a major cashmere producer (Mongolia is the world’s second largest cashmere producer behind China), it produces about 30% of the world’s cashmere [19]. There are eight goat strains/breeds recognized in Mongolia [20,21], and they are adapted well to various natural zones including forest, steppe, desert, and high-mountain. Unfortunately, little is known about the genetic diversity, phylogenetic relationships, and origins of goat populations in Mongolia. Only a few samples were included in merged datasets of previous studies that explored the population structure of world goat populations [6,14,22]. We therefore initiated a study of partial mtDNA sequences to evaluate the genetic diversity and origin of Mongolian native goat (MG). Then, the main objective of this study was to assess the genetic diversity, phylogenetic status and population structure of MG, as well as to discuss their origin together with other foreign breeds from different countries using hypervariable region 1 (HV1) in mtDNA.

## MATERIALS AND METHODS

### Animal care

All sampling procedures in this study were carried out in accordance with the recommendations of “Guide for Care and Use of Agricultural Animals in Research and Teaching, 3rd edition” published by the Federation of Animal Science Societies (ISBN: 978-1-884706-11-0) and also “Guidelines for sampling and handling animals for purposes of Veterinary and Other researches” of Livestock sector of Mongolia, with full approval from Committee Ministry of Agriculture and Food of Mongolia (ref. A67/2010, in Mongolian). The protocol approved by Committee of Ministry of Agriculture and Food of Mongolia was used in this study. Animal owners had informed consent for inclusion of their animals in the study, and they involved sampling procedures.

### Animals and DNA extraction

**Figure 1:**
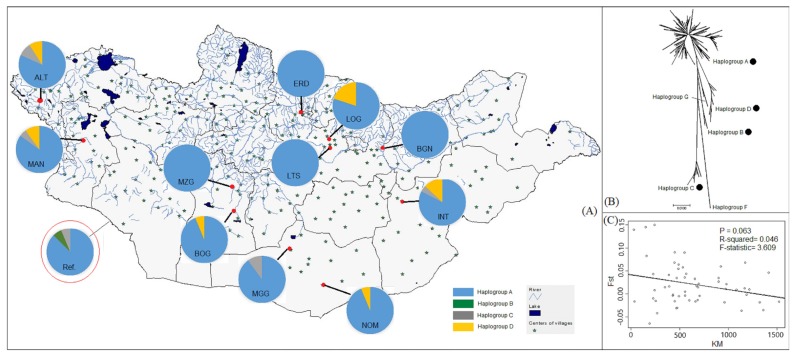
(A) Sampling localities of 12 Mongolian native goat (MG) populations (population ID presented): red spots indicating exact sampling locations, Ref. population located outside without specific location. (B) Un-rooted neighbor-joining tree that illustrating four haploroups of MG (black spots). And (C) Regression plot of relationships of genetic (*Fst*) and geographic (km) distances.

Sampling was conducted across Mongolian territory in 2017 and 2018. In this study, a total of 158 unrelated genetic samples that consisted of 97 blood (2017), 10 ear skin, and 51 tail hair (2018) were gathered from 11 different populations (Figure 1A). We collected different samples during sampling years due to availability of field equipment. Of 11 populations, two populations were recognized as separate native strains (Zalaa Jinstiin White; MZG and Gobi-Gurwan Saikhan Black; MGG), while the other nine populations were believed to be Mongolian indigenous native goats (MNG) (Table 1, Figure 1A). Geographical distances between sampling sites ranged from ~65 km to ~1,450 km. When selecting animals from each investigated population, we involved owners to avoid sampling from related animals. Genomic DNA was extracted from punches of filter paper (FTA classic cards Whatman: Lot No.9767339) using a MagMax DNA Multi-Sample Ultra Kit, while genomic DNA was extracted from ear tissue and hair samples using a DNeasy Blood and Tissue Kit (QIAGEN, Hidden, Germany) following the recommended manufacturer’s instructions.

### Polymerase chain reaction amplification and sequencing

To study genetic diversity and phylogenetic analyses of MG, we amplified a 452 bp mtDNA fragment of the caprine partial HV1 region in D-loop (positions 15,678 to 16,130 on the complete goat mitochondrial sequence of reference NC_ 005044) from158 animals using a set of primers: gd-loop F (5′-CAC AAA CTT CCC ACT CCA CA -3′) and gd-loop R (5′-AGC GTG TTT AAA ACG GTG GT -3′). We designed these two internal primers using the online tool Primer3Plus (online available at: http://www.bioinformatics.nl/cgi-bin/primer3plus/primer3plus.cgi) based on a reference sequence (accession number: NC_005044). Polymerase chain reaction (PCR) amplifications were performed on a total of 20 μL final volume that included 2.0 μL genomic DNA (20 ng/μL), 0.8 μL of each primer (10 pmoles/μL), 2.0 μL 10× reaction buffer, 1.6 μL dNTP, 0.2 μL prime Taq DNA polymerase, and 12.6 μL distilled water to adjust the final PCR product volume. The reaction conditions for amplification consisted of initial denaturation at 94°C for 3 min, followed by 35 cycles with denaturation for 30 s at 94°C, annealing at 55°C for 30 s, extension for 1 min at 72°C, and final extension at 72°C for 5 min. All PCRs ran with negative controls and were performed by a thermal cycler C1000 system (Bio-Rad laboratories Inc., Hercules, CA, USA). After amplification, approximately 5 μL of PCR product was run on 1.5% agarose gel at a constant voltage of 150 V for 30 min in 1×TAE buffer prior bands and size checked under UV light. A service provider company (GenoTech Corp. Deajeon, Korea) performed direct sequencing of purified PCR products using a Sanger DNA sequencing approach in an ABI Prime 3730 DNA sequence machine. All 158 novel sequences of Mongolian goats in this study were deposited in the Genbank public database under accession numbers MK165491–MK165648.

### Data assemble and analysis

A total of 158 sequences representing 11 MG populations from this study were merged with the previously described 16 sequences (considered as separated populations) for total of 12 MG populations. To examine the phylogenetic status and the origin of MG, 174 sequences of Mongolian goats were initially edited with BioEdit v.7.2.5 [23] and partial sequences aligned positions using the ClustalW tool [24] together with an additional 329 reference sequences of 24 foreign breeds from different geographic regions in the Old World and three wild *caprine* species, as well as representative sequences of the previously defined six *caprine* haplogroups (A, B, C, D, F, and G) (accession numbers of all reference sequences in Supplementary Table S1).

At the individual population level, as well as all MG together, DNA Sequence Polymorphism v5.10.01 (DnaSP [25]) was used to calculate the following diversity parameters of mtDNA: number of haplotypes (h), haplotype diversity (*H*d), number of segregating sites (S), nucleotide diversity (Pi), and average number of nucleotide differences (K).

We constructed a neighbor-joining (NJ) tree (using 500 bootstraps to determine evolutionary distances computed based on the Tamura-Nei distance model; [26]) to examine possible haplogroups in MG populations and phylogenetic relationships among MG populations, as well as other foreign breeds using Molecular Evolutionary Genetic Analysis version.7.0.21 (MEGA [27]). The genetic distance (Tamura-Nei) between 12 MG populations were also assessed using MEGA. The Roehl network data (*.rdf) files for network analysis were generated by DnaSP. Then, we drew the Median-Joining (MJ) network using NETWORK v.5.0.0.1 [28] to trace genetic relationships among identified haplotypes within MGs and between other breeds from different regions.

Arlequin v3.5 [29] was used to estimate pairwise genetic distance (significant values were accepted when p<0.05), average number of pairwise differences between and within populations, and analysis of molecular variance (AMOVA) to assess the population genetic structure (with 1,000 permutations). We used, and explored in Arlequin v3.5, two main approaches: i) Fu’s (1997) neutrality tests of *Fs* and ii) mismatch distribution analysis [30], to assess signs of historical population expansions. Two tests of goodness of fit, sum of squared deviation (SSD) and Harpending’s raggedness index (r) [31], were used to evaluate the significance of deviations within observed patterns of mismatch from the simulated model of demographic expansion (p value accepted at <0.05).

## RESULTS

### mtDNA variation and genetic diversity

Comparison of 174 sequences of the HVI region of goats from 12 different Mongolian goat populations showed high polymorphism. There were 109 polymorphic sites over 452 bp, while only two insertion/deletion (InDel) mutations were detected. Estimated diversity parameters for MG populations are given in Table 1. Among these polymorphic sites, we identified 20 singletons and 89 parsimony informative sites that defined 137 haplotypes (three of these haplotypes defined by two indels) with a relatively high *H*d (overall; 0.997±0.001). Estimated values of *H*d ranged from 0.933 (Erdenet; ERD) to the maximum value of 1.000 (MGG, MZG, and Ref), while nucleotide diversity (Pi) varied between 0.0199 (Lun Tsag; LTS) and 0.0384 (Altai; ALT) (overall, Pi = 0.0283) (Table 1). The mean K was 12.75, and the highest K-value was observed for the ALT population (17.34). In addition, an average base composition was A = 31.0%, T = 30.3%, G = 16.5%, and C = 22.2% in a 452 bp HVI region. Thus, the percentage of A+T (61.3%) nucleotide pairs was higher than the nucleotide pairs of C+G (38.7%).

### Phylogeography of Mongolian goats

**Figure 2:**
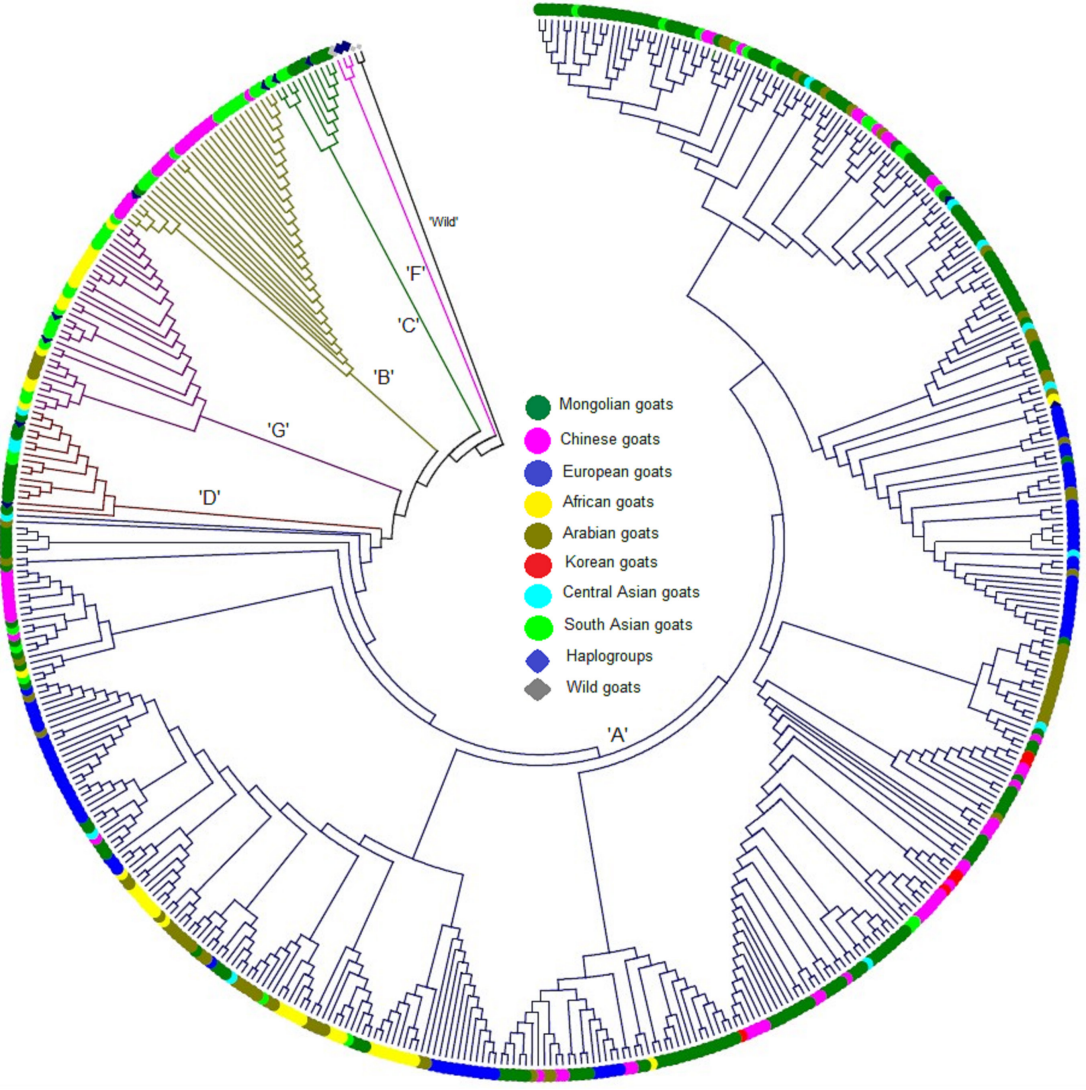
Constructed neighbor-joining tree of domestic (*Capra hircus*) and wild (genus of *Capra*) goats based on 452 bp hypervariable region 1 region from 503 sequences (174 MG and 329 reference sequences from different regions).

Phylogenetic relationships of goat populations within Mongolian and between 36 breeds (MG plus foreign breeds) were initially investigated in this study based on the high polymorphic HVI region in mtDNA. Because previous studies [6,11] have described two origins for haplogroups of domestic goat (one for B, remaining one same for other five haplogroups), haplogroups in MG were identified through an un-rooted NJ tree using representative sequences of six haplogroups to discern possible origin for MG in this study. More in detail, the NJ tree clearly divided all MG sequences into four different haplogroups: A, B, C, and D (Figure 1B). The findings suggested two origins for the MG population. Among these, haplogroup A was highly predominant and found in all populations of MG (100% for ERD, LTS, Baganuur; BAG, and MZG). In contrast, relatively low numbers of animals belonged to haplogroups C (6/12 populations) and D (5/12), and only one for haplogroup B (1/12) (Table 1, Supplementary Table S2). Thus, the frequencies of haplogroups A, B, C, and D in MG populations were 90.8%, 0.57%, 3.4%, and 5.2%, respectively. Furthermore, ALT, Ikh Nart (INT), and Mankhan (MAN) populations were found to contain three haplogroups (A, C, and D), while there was no population with all four haplogroups (Table 1). To obtain further insight into the phylogenetic status of MG, a NJ tree was constructed once again for 12 MG populations and a total of 503 sequences that consisted of 36 populations (24 foreign and 12 MG), as well as three species of wild caprine using Tamura-Nei distance model (Figure 2, Supplementary Figure S1). Supplementary Table S3 presents Tamura-Nei genetic distance matrix (within/between) of MG. The results of NJ tree and genetic distance matrix showed relatively close relationships of MG populations, and indicated an absence of population-specific clusters in the NJ tree (Supplementary Figure S1). In addition, goats from China, Korea, and Central Asian (Kazakh and Kyrgyz), and some from Arabia had relatively close relationships compared to MG, while goats from Europe, Africa, and South Asia (except some Indian goats) clustered separately (Figure 2). Among the MG populations, Tamura-Nei distance within population reached a maximum estimated value in ALT (0.0402), while LTS accounted for the lowest value (0.0204). The genetic distance values between populations varied between 0.0389 (LOG versus Ref.) and 0.0207 (LTS vs BGN) (mean value was 0.0302) (further information see Supplementary Table S3).

**Figure 3:**
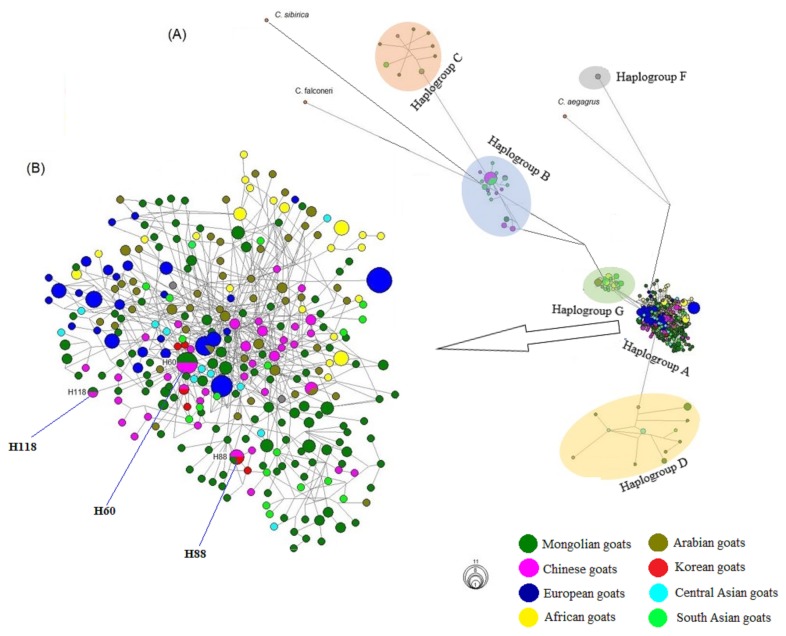
Median-joining network analysis of hypervariable region 1 region from native goats (*Capra hircus*) from Mongolia (populations, n = 12), and foreign countries (populations, n = 24). (A) network topology of six caprine haplogroups, and (B) predominant haplogroup A with highlighted three haplotypes that shared by Mongolian native goat and other foreign breeds.

To further discern relationships among identified haplotypes in MG populations and in the total dataset (503 sequences), a MJ network analysis was constructed separately. Results obtained from this analysis also supported four well-resolved haplogroups of MG (Figure 3). The topology of the MJ network illustrated relationships of 355 haplotypes of 503 sequences. As mentioned above, 137 of 355 haplotypes belonged to MG, and the majority of these 137 haplotypes clustered into haplogroup A (160 total haplotypes), with only one haplotype for haplogroup B, six haplotypes in haplogroup C, and seven haplotypes in haplogroup D (Figures 1B, 3). Among MG, 121 of 137 haplotypes were unique for a single population, whereas only 16 (11.6%) haplotypes were shared between two or three MG populations, and H19 and H60 were observed as the most frequent haplotypes with four animals (Figure 3, Supplementary Tables S2, S4). In addition, among 355 haplotypes, the three haplotypes that clustered into haplogroup A (H60, H88, and H188) were shared by Mongolian goats and goats from China (seven goats) and Korea (one goat) (Figure 3). Interestingly, Figure 3 also revealed lack of haplotypes that shared between multiple populations or breeds from different regions.

### Genetic differentiation and population structure

Additionally, we calculated Wright’s F-statistic (pairwise *Fst*) and average number of pairwise differences between (PiXY) and within (PiX) population values among 12 MG populations, while we also calculated Fst values for comparison among all 36 populations. Estimates of values of genetic differences are indicated in Tables 2 and Supplementary Table S5, respectively. The pairwise *Fst* values between 12 MG populations revealed relatively low genetic differentiation and ranged between −0.065 and 0.150 (mean *Fst* = 0.0168). The MGG population was closely related genetically to Nomgon (NOM; −0.065), followed by the distance between NOM and Ref (−0.0543), while comparison of LOG and BGN populations had a greater distance value (0.150) (Table 2). There were no significant relationships between genetic (*Fst*) and geographical (km) distances among MG populations (R^2^ = 0.04, d.f = 53, p>0.05) (Figure 1C). Table 2 also presents the average number of pairwise differences between and within MG populations, and mean values were relatively similar, 13.8 and 13.6, respectively. The maximum PiXY value was obtained from a comparison between LOG versus Ref (18.08), and the minimum value was observed between LTS and BGN (9.326). Meanwhile, PiX values ranged from 18.44 (ALT) to 9.21 (LTS). The low genetic differentiation of MG populations was also evidenced by AMOVA analysis in this study. AMOVA revealed poor geographical structure among populations, 98% of total variation was within populations and only <2% was due to differences among populations (Table 3).

In addition, pairwise *Fst* values from comparisons of 36 breeds from MG and foreign countries and the majority of all possible pairwise *Fst* comparisons (234 of 288) indicated that MG populations were significantly (p<0.05) different from foreign populations, in particularly those from Africa, Europe, and some of southern Asia (Iran and Loas) (Supplementary Table S5). However, some populations were closely related to MG populations, in particularly a negative *Fst* value was observed between Kazakh and Mongolian goats (−0.190), followed by low positive values 0.026 for Indian and MGs, and goats from Arabian countries (including the domestication center).

### Population expansion

**Figure 4:**
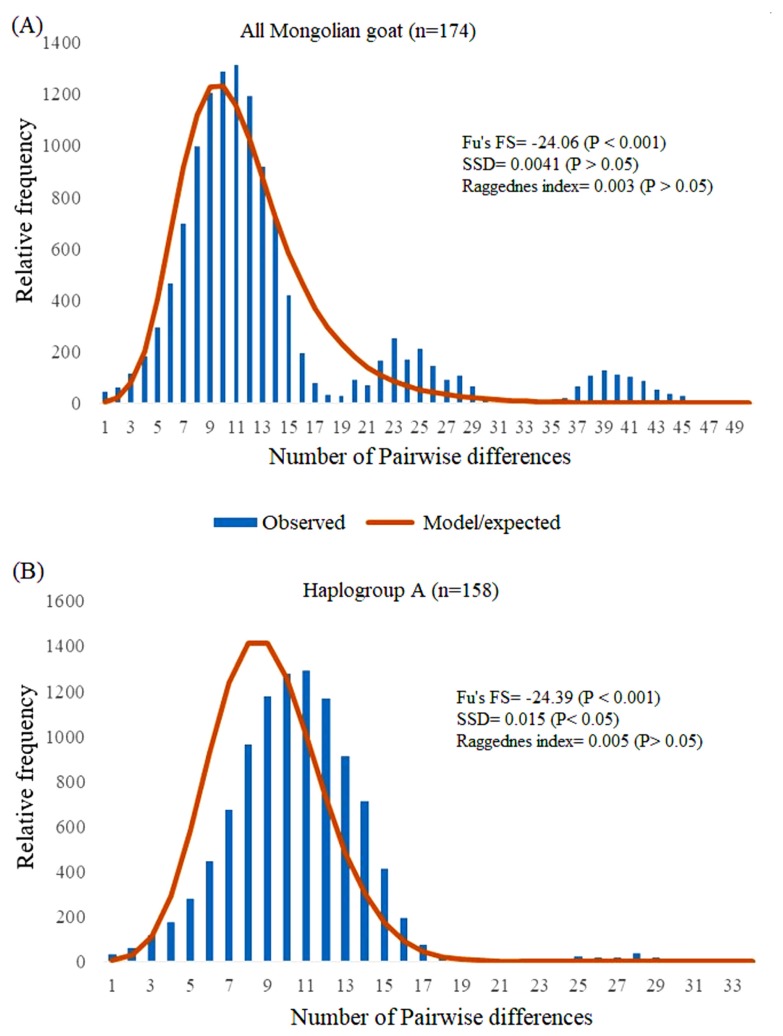
Mismatch distributions and Fu’s *Fs* analysis of (A) all Mongolian goats (n = 174) and (B) haplogroup A (n = 158).

We initially studied past demographic dynamics of MG based on two standard approaches of mismatch distribution (number of pairwise differences) and the neutrality test of Fu’s [32] *Fs* statistic. Because of the small sample size at the individual breed level (<25), these statistics were performed for all datasets of MG and at the haplogroup level (but, haplogroups B, C, and D were precluded due to small sample sizes). All MG and haplogroup A displayed multimodal and unimodal curves that mismatched distributions, respectively (Figure 4). In particularly, all MG showed two major curves at 11 and 23 pairwise differences with one minor curve at 39 pairwise differences. These results indicate there were at least two population expansion events that occurred in the past. Fu’s [32] *Fs* statistic provided more evidence for past population expansion of MG; this value was significantly negative for all MG and haplogroup A (p<0.001) (Figure 4). In addition, SSD and r values were not significant for all MG goats (p>0.05; 0.004 and 0.003, respectively), indicating that our total dataset (174 MG) showed relatively good fit to the population expansion model [31].

## DISCUSSION

### Genetic diversity

To date, no study has explored genetic diversity and the origin of MGs in depth. This study conducted the first assessments of genetic population structure, genetic diversity, phylogenetic status, and the origin of Mongolian goats using the most polymorphic HVI region in mtDNA. This highly polymorphic region (indeed, the complete D-loop) has been routinely used in previous studies that examined genetic diversity, phylogenetic relationships, and especially the maternal origin of most domesticated animals, including goats [14,33–37].

Similar to previous results [17,38,39], goats from Mongolia investigated in this study showed high mtDNA diversity. The assessed overall *H*d and Pi of MG were 0.997 and 0.0283, respectively (Table 1). Our results also indicated that *H*d in Mongolian goats were slightly higher (not significant) than that found in goat breeds from Africa, Egypt, and south Asia, but significantly higher than in Chinese and European breeds (Supplementary Figure S2A) [17,38,39]. Whereas, Pi values obtained from this study were significantly higher than that found in other regions including domestication center (except Chinese and southern Asian breeds) (Supplementary Figure S2B) [17,38,39]. The high level of mtDNA diversity in the world’s goat populations was previously explained by multiple maternal effects of wild ancestors [5,6]. Similarly, the high genetic diversity in MG likely resulted from the presence of an abundant haplogroup and crossbred/mixed with foreign populations from the Union of Soviet Socialist Republic (USSR) (Mountain Altai, Don and Unjuul) to improve the quality of their fibers [20].

### Maternal origin and phylogeography of Mongolian goats

As the origin of MG was only initially investigated in this study, these results are far from clear. Mongolians traditionally believed that their goats descended directly from wild Siberian ibex, a species widely distributed in Mongolia [19]. Yet, genetic and archeological studies widely agree that domestic goats in Mongolia originated from *C. aegagrus* in Fertile Crescent region of the NE [1,2,6,7]. Meanwhile, Lin et al [22] emphasized that haplogroup B or at least sub-group B1 originated in some region of Southeast Asia. Therefore, all animals that clustered into haplogroup B belong to east or south Asian countries, in particularly sub-group B1 accounted for only Chinese and Mongolian goats in this study (Figures 1B, 3).

The results of our phylogenetic analysis revealed four distinct maternal haplogroups (A, B, C, and D) in MG populations (Figure 1B). Consistent with previous studies [6,14,15,22,40], haplogroup A was predominant (90.8%) and found in all MG populations. Haplogroup B was found mostly in Asia, but only one individual from the Ref population was found in this haplogroup in this study (Figure 3). Alternatively, haplogroups C and D were found from both Asia and Europe, and goats from G and F had very limited distributions in Sicily and the NE region, respectively [6]. Some previous studies [6,14] reported that three haplogroups (A, B, and C) occurred in Mongolia based on a very limited sample size. More recently, Lin et al [22] included some Mongolian goats (population’s locality unknown) in their diversity analysis. They reported the four haplogroups that we found in this study. To obtain further insight into the origin of MG, NJ tree, MJ network, and estimation of pairwise *Fst* values were performed based on a combination of goats from Mongolia and those from different regions, including the domestication center (Figures 2, 3). The close relationships of Mongolian and Chinese (from Huanghuai and Leizhou) goats were obtained from MJ network analysis; they shared three haplotypes (H60, H88, H118, Figure 3, Supplementary Table S4) and occurred in NJ trees’ clusters. Interestingly, goat populations (n = 1) from Kazakhstan were very closely related genetically to MG (mean, *Fst* = −0.016), and followed by *Fst* values (mean = 0.103) obtained from comparisons of goat populations (n = 5) from some Arabian countries (including the domestication center; i.e. Turkish goat and Baladi breed from Jordan) and MG populations (n = 12), this value was lower than that found in comparisons of Chinese (n = 5) versus MG populations (0.128), as well as southern Asia (n = 4, mean *Fst* = 0.275) (Supplementary Table S5). These close relationships among goats from different regions likely resulted from a weak phylogeographic structure of domestic goats, which is likely explained by a high rate of gene flow among populations, even between continents [38].

According to findings from this and previous studies [6,14, 22,39], goats from the four haplogroups in Mongolia arrived from two possible domestication centers through ancient human migration or commercial trade routes. We hypothesize that the first domestication center was the NE region for MG from haplogroups A, C, and D whose ancient migration routes could be explained by a migration that started in the NE region and passed through central Asia (i.e. Kyrgyzstan and Kazakhstan, *Fst* values versus MG was 0.106 and −0.016, respectively), prior to arriving in Mongolia. This migration may have involved ancient sheep population, because since ancient times Mongolian nomads reared sheep and goat together.

Second, a domestication center was located close to Mongolia somewhere in Southeast Asia [22]. We believe that a smaller group of ancient goats from haplogroup B (B1 subgroup) arrived in Mongolia from this center. It is difficult to determine the date of the first arrival of domestic goats into Mongolia. Nevertheless, archeological findings based on petroglyphs in Mongolia demonstrate that the first goat populations arrived at least 3,000 to 3,500 years ago [6,41]. If the first goats arrived with sheep in Mongolia, this dated could be pushed back to 5,000 to 7,000 years ago [10].

### Population structure and expansion

AMOVA analysis of all MG dataset showed very weak phylogeographic structure among 12 populations, and more than 98% of the total variation occurred within populations. This weak structure was also evidenced by limited genetic differentiations (mean pairwise *Fst* = 0.0168). Our results were consistent with previous studies [6,38,42]. The traditional nomadic culture of Mongolia may have contributed to this weak geographical structure and limited genetic differentiations among MG populations. Under nomadic pasturing, there is a high rate of animal exchanges of the five main types of livestock animals in Mongolia (sheep, horse, cattle, camel, and goat) and often long-distance movements even cross the country.

Mismatch distribution and the neutrality test of Fu’s [33] *Fs* statistics are recognized as suitable approaches to examine past population expansion and were routinely used in previous studies [6,36,38,39]. Mismatch distribution is a frequency graph of pairwise differences between alleles, and it has two common patterns, including multimodal (populations at demographic equilibrium) and unimodal (populations passed through a recent demographic expansion) [29,43]. Fu’s *Fs* statistic is based on the probability of having a number of alleles equal to or greater than the observed number in a sample drawn from a stationary population [32]. Both approaches revealed at least two population expansions of Mongolian goats in the past: i.e. mismatch distribution pattern was multimodal for all MG while unimodal for haplogroup A, and highly significant and negative Fu’s *Fs* values were observed for all MG (−24.06) and haplogroup A (−24.39). Relatively similar patterns of mismatch distribution and significant and negative Fu’s *Fs* values were obtained in previous studies [37, 39,42]. More recently, the number of goats in Mongolia has increased fivefold within the last 30 years, more specifically the goat population grew from 5.1 million in 1990 to 27.3 million by 2017 [18].

## CONCLUSION

In summary, we report novel information regarding genetic diversity, population structure, and origin of Mongolian goats. The findings obtained from this study reveal that abundant haplogroups (A to D) occur in goat populations in Mongolia, with high levels of haplotype and Pi. Among these haplogroups, haplogroup A was predominant, with relative frequency of 90.8%. In addition, we speculate that two independent domestication centers may have contributed to the formation of goat populations in Mongolia. Finally, we highlighted that the past nomadic pasturing system led to weak geographical structure and limited genetic differentiations among populations in different regions of Mongolia.

## Supplementary Data



## Figures and Tables

**Table 1:** Estimated genetic diversities in Mongolian native goat (*Capra hircus*) populations and their haplogroup distributions

Populations	ID	n1)	h2)	*H*d3)	S4)	Pi5)	K6)	H7)(n)
Altai	ALT	11	10	0.982±0.046	65	0.0384±0.009	17.34	A(9), C(1), D(1)
Baganur	BGN	20	19	0.995±0.018	41	0.0201±0.001	9.068	A(20)
Bogd	BOG	16	14	0.983±0.028	39	0.0213±0.004	9.608	A(15), D(1)
Erdenet	ERD	10	7	0.933±0.062	31	0.0217±0.003	9.800	A(10)
Ikh Nart	INT	23	21	0.992±0.015	69	0.0319±0.005	14.371	A(19), C(1), D(3)
Mankhan	MAN	20	18	0.989±0.019	69	0.0329±0.006	14.563	A(17), C(1), D(2)
Lun Og	LOG	10	9	0.978±0.054	42	0.0355±0.004	16.067	A(8), D(2)
Lun Tsag	LTS	18	13	0.961±0.030	36	0.0199±0.001	8.993	A(18)
Nomgon	NOM	10	8	0.956±0.059	53	0.0334±0.009	15.089	A(9), C(1)
Bulgan	MGG	10	10	1.0±0.045	53	0.0321±0.011	14.533	A(9), C(1)
Jinst	MZG	10	10	1.0±0.045	31	0.0211±0.001	9.533	A(10)
Ref.	Ref. 8)	16	16	1.0±0.022	72	0.0368±0.007	16.63	A(14), B(1), C(1)
Overall	12	174	137	0.997±0.001	109	0.0283±0.002	12.75	A(158), B(1), C(6), D(9)

1) Number of animals.

2) Number of haplotypes.

3) Haplotype diversity.

4) Number of segregating sites.

5) Nucleotide diversity.

6) Average number of nucleotide differences.

7) Identified haplogroups in each population (number of animals).

8) Animals from Luikart et al [14] as a reference population.

**Table 2:** Estimates of pairwise *Fst* (below) and average number of pairwise differences between (PiXY, above) and within population (PiX, diagonal) among 12 native Mongolian goat (*Capra hircus*) populations

Items	INT	LTS	LOG	ERD	MGG	MZG	NOM	BOG	MAN	BGN	ALT	Ref.
INT	**15.429**	12.908	16.369	13.753	15.415	12.971	15.237	12.957	15.108	12.948	16.587	16.283
LTS	0.041*	**9.212**	14.796	10.480	12.222	9.330	12.505	9.897	12.564	9.327	14.586	13.769
LOG	0.016	0.140*	**16.872**	15.766	17.409	14.648	17.188	14.109	16.241	14.955	17.886	18.088
ERD	0.058*	0.083*	0.146*	**10.067**	13.290	10.492	13.329	10.971	13.334	9.800	14.434	14.373
MGG	−0.005	−0.002	0.068*	0.036	**15.566**	12.157	14.825	12.446	15.172	12.222	16.984	16.064
MZG	0.015	−0.016	0.091*	0.055	−0.042	**9.766**	12.495	9.682	12.519	9.364	14.539	13.773
NOM	−0.031	0.005	0.044	0.022	−0.065	−0.031	**16.006**	12.794	15.111	12.312	16.644	16.004
BOG	0.017	0.034	0.064	0.090*	−0.015	−0.016	−0.004	**9.907**	12.606	9.920	14.895	13.944
MAN	−0.018	0.021	0.011	0.035	−0.018	−0.014	−0.036	−0.004	**15.344**	12.440	16.672	16.199
BGN	0.043*	0.008	0.150*	0.015	−0.004	−0.016	−0.014	0.033	0.010	**9.289**	14.280	13.566
ALT	−0.017	0.068*	0.012	0.009	−0.002	0.027	−0.036	0.060*	−0.010	0.046*	**18.447**	17.706
Ref.1)	−0.016	0.026	0.043	0.023	−0.037	−0.008	−0.054	0.010	−0.019	0.010	−0.020	**17.694**

1) Animals from Luikart et al [14] and considered Ref. population in this study.

* Indicates significant Fst (p<0.05).

**Table 3:** Genetic variance of Mongolian native goats (*Capra hircus*) based on analysis of the hypervariable region 1 region

Source of variation	Degree of freedom	Sum of square	Variance component	Percentage of variation
Among population	11	85.73	0.095	1.46
Within population	162	1,040.98	6.425	98.54
Total	173	1,126.71	6.520	-

*Fst* = 0.014, p<0.05.
