# Supplementary material for: Mitochondrial DNA variation and phylogeography of native Mongolian goats
Source: Asian-Australas J Anim Sci. 2019 Oct 21;33(6):902–12. doi: 10.5713/ajas.19.0396 (PMC7206382; doi:10.5713/ajas.19.0396)
Supplement: Supplementary file 1 [file ajas-19-0396-suppl.pdf]

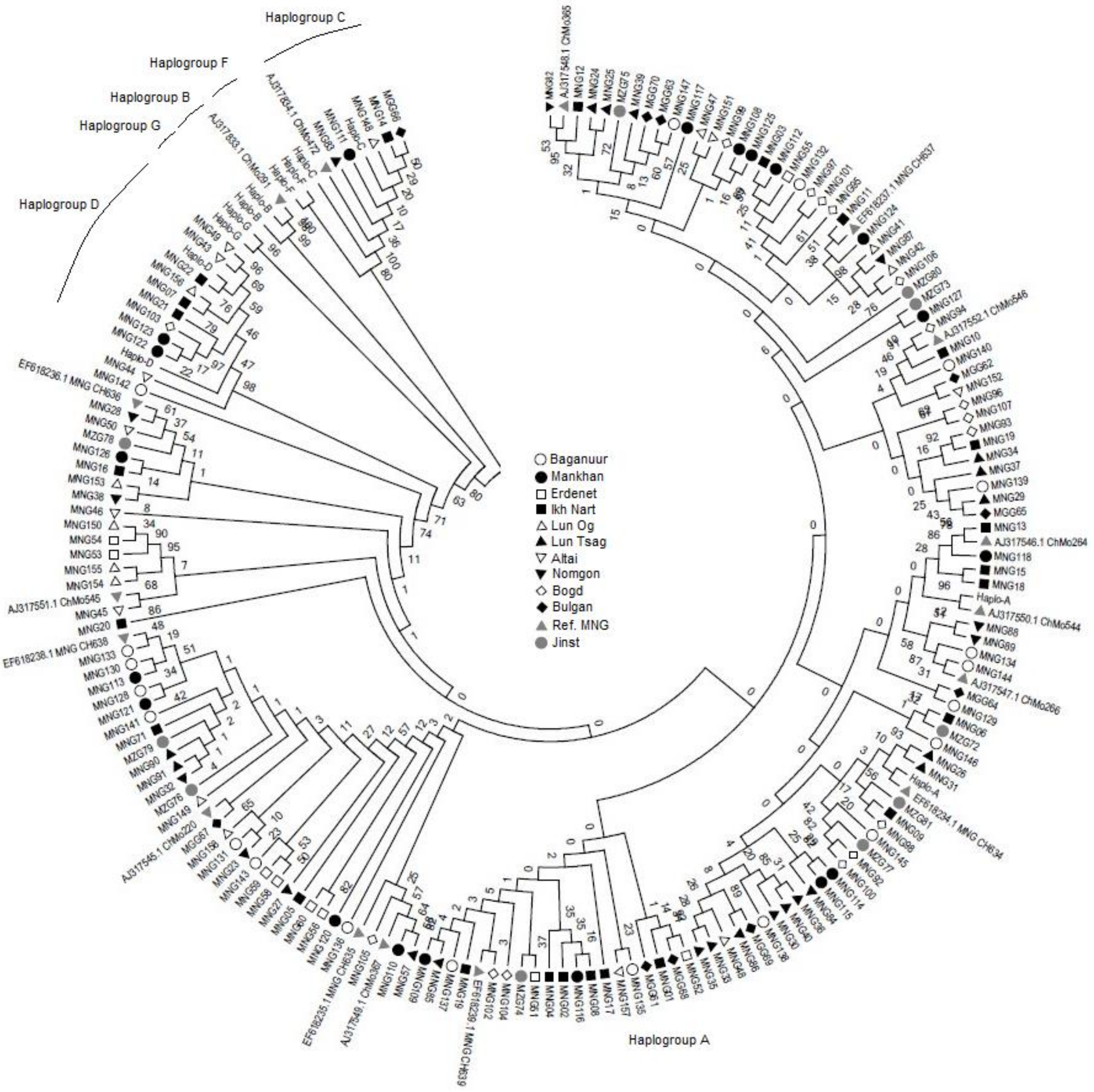

Supplementary Figure S1. Neighbor-joining (NJ) trees for 12 MG populations

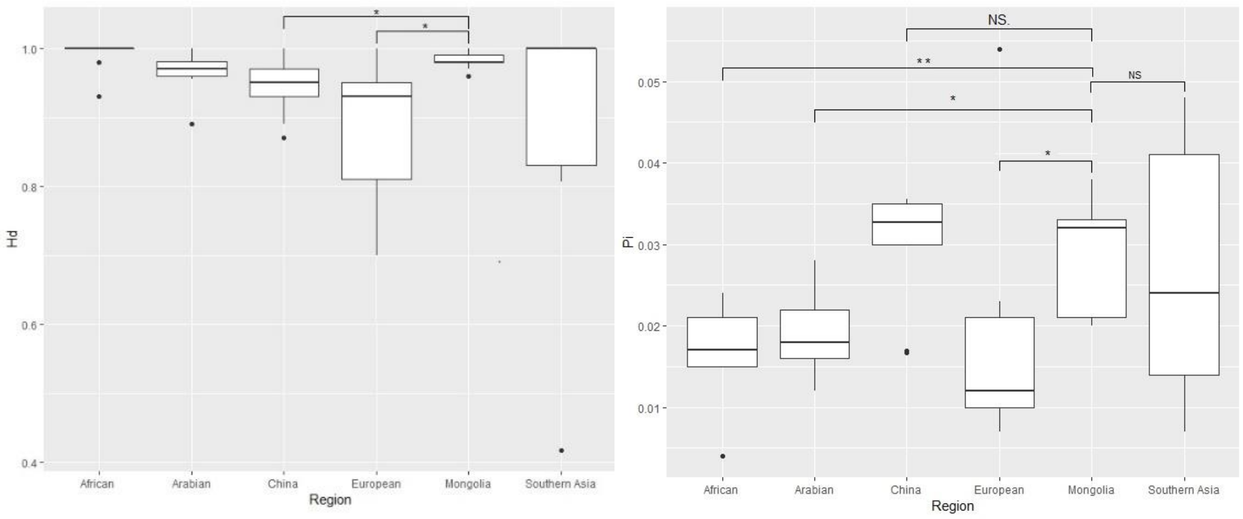

Supplementary Figure S2. Boxplot for comparisons of haplotype (*H*d, left) and nucleotide diversity (Pi, right) of goat populations from different geographical regions (using R packages “ggplot2” and “ggsignif”). Haplotype and nucleotide diversity values were calculated based on same 452 bp long HVI region for all populations from different region.

Supplementary Table S1. Accession numbers and other information of reference sequences that retrieved from Genbank for this study.

Breeds	ID	No. of samples	Ref.	Region	Accession numbers
Gondar	GO	20	Tarekegn et al., 2017	African	KY747819, KY747841, KY747847, KY747849, KY747859, KY747873, KY747879, KY747882, KY747890, KY747901, KY747918, KY747920, KY747922, KY747968, KY747973-75, KY747977, KY747989, KY747993
Kenyan native goat	KG	21	Kibegwa et al., 2016	African	KP120622- KP120642
Turkey goat	TG	15	Luikart et al., 2001	Arabian	AJ317737- AJ317751
Baladi	Ba	14	Naderi et al., 2007	Arabian	EF618191- EF618204
Batinah	Bat	12	Othmen et al., 2016	Arabian	KY399058- KY399069
Beeshi	Be	15	Naderi et al., 2007	Arabian	EF618309- EF618330
Arbi	Ar	10	Vacca et al.., 2010	Arabian	FJ571562- FJ571571
Chengduma	Che	13	Ruo et al., 2006	China	DQ121506- DQ121518
Guizhou black	GuB	12	Ruo et al., 2006	China	DQ121521- DQ121532
Huanghuai	Hua	12	Ruo et al., 2006	China	DQ121550- DQ121561
Leizhou	Lei	13	Ruo et al., 2006	China	DQ121564- DQ121576
Shaanen white	SNW	15	Ruo et al., 2006	China	DQ121604- DQ121618
Korean native goat	KNG	6	Odahara et al., 2006	East Asian	DQ217780- DQ217785
Mongolian native goat	Ref.	16	Luikart et al., 2001	East Asian	AJ317833- AJ317552
Majorera	MJ	21	Amillis et al., 2004	European	AY424919, AY424921-22, AY424928, AY424933, AY424940-43, AY424949, KM893157, KM893159, KM893230, KM893233-34, KM893236, KM893241, KM893243, KM893293, KM893308-10.
Derivata di Siria	DdS	10	Sardina et al., 2006	European	DQ241363- DQ241371
Gergentana	Ger	16	Sardina et al., 2006	European	DQ241332- DQ241347
Maltese	ML	11	Sardina et al., 2006	European	DQ241352- DQ241362
Pinalera	Pin	13	Ferrando et al., 2015	European	KM893154-56, KM893306, KM893200-17
Indian native goat	IG	12	Joshi et al., 2004	Southern Asia	AY155681-85, AY155707-09, AY155834-36, AY155876-82, AY155952-54, AY156000-01
Iran native goat	ING	15	Naderi et al., 2007	Southern Asia	EF618070- EF618084
Loas native goat	LOG	10	Mannen et al., 2001	Southern Asia	AB044295- AB044304
Pakistan goats-1	PG	14	Sultana et al., 2003	Southern Asia	AB110553-64, AB110571, AB110587, AB110589
Kazakstan goat	Kag	6	Naderi et al., 2007	Central Asian	EF618205- EF618210
Kyrgyz goat	Kyg	8	Naderi et al., 2007	Central Asian	EF618212- EF618219
Haplogroup A	HA	1	AY155721	-	
Haplogroup A	HA	1	EF618134	-	
Haplogroup B	HB	1	AB044303	-	
Haplogroup B	HB	1	AJ317833	-	
Haplogroup C	HC	1	EF618413	-	
Haplogroup C	HC	1	AY155708	-	
Haplogroup D	HD	1	DQ188893	-	
Haplogroup D	HD	1	AY155952	-	
Haplogroup G	HG	1	EF618084	-	
Haplogroup G	HG	1	EF618535	-	
Haplogroup F	HF	1	DQ241349	-	
Haplogroup F	HF	1	DQ241351	-	
C. aegagrus	-	1	AJ317864	-	
C. sibirica	-	1	AJ317874	-	
C. falconeri	-	1	AB044305	-	
	**36**	**503**			

Supplementary Table S2. Distribution of haplotypes among 12 Mongolian native goat populations

Hap	INT	LTS	LOG	ERD	MGG	MZG	NOM	BOG	MAN	BGN	ALT	Ref	
**h1**	1												**1**
**h2**	2								1				**3**
**h3**	1								1				**2**
**h4**	1												**1**
**h5**	1												**1**
**h6**	1												**1**
**h7**	1												**1**
**h8**	1												**1**
**h9**	1												**1**
**h10**	1												**1**
**h11**	1												**1**
**h12**	1											1	**2**
**h13**	1												**1**
**h14**	2												**2**
**h15**	1												**1**
**h16**	1												**1**
**h17**	1									1			**2**
**h18**	1												**1**
**h19**	1							1	2				**4**
**h20**	1												**1**
**h21**		1											**1**
**h22**		2											**2**
**h23**		2											**2**
**h24**		1											**1**
**h25**		1											**1**
**h26**		1											**1**
**h27**		3											**3**
**h28**		1											**1**
**h29**		2											**2**
**h30**		1											**1**
**h31**		1											**1**
**h32**		1											**1**
**h33**		1											**1**
**h34**			1				1						**2**
**h35**			1					1					**2**
**h36**			2										**2**
**h37**			1										**1**
**h38**			1									1	**2**
**h39**			1										**1**
**h40**			1										**1**
**h41**			1										**1**
**h42**			1										**1**
**h43**				1									**1**
**h44**				1									**1**
**h45**				2							1		**3**
**h46**				1									**1**
**h47**				2									**2**
**h48**				1									**1**
**h49**				2									**2**
**h50**					1								**1**
**h51**					1								**1**
**h52**					1					1			**2**
**h53**					1								**1**

**h54**					1								**1**
**h55**					1								**1**
**h56**					1							1	**2**
**h57**					1								**1**
**h58**					1		1						**2**
**h59**					1								**1**
**h60**	1					1	2						**4**
**h61**						1							**1**
**h62**						1							**1**
**h63**						1							**1**
**h64**						1							**1**
**h65**						1							**1**
**h66**						1		2					**3**
**h67**						1							**1**
**h68**						1							**1**
**h69**						1							**1**
**h70**							1						**1**
**h71**							1						**1**
**h72**							1						**1**
**h73**							1						**1**
**h74**							2						**2**
**h75**								1	1				**2**
**h76**								1					**1**
**h77**								1					**1**
**h78**								2					**2**
**h79**								1					**1**
**h80**								1					**1**
**h81**								1					**1**
**h82**								1					**1**
**h83**								1					**1**
**h84**								1					**1**
**h85**								1					**1**
**h86**									1				**1**
**h87**									1				**1**
**h88**									1				**1**
**h89**									1				**1**
**h90**									1				**1**
**h91**									2				**2**
**h92**									1				**1**
**h93**									1				**1**
**h94**									1				**1**
**h95**									1				**1**
**h96**									1				**1**
**h97**									1				**1**
**h98**									1				**1**
**h99**									1				**1**
**h100**										1			**1**
**h101**										1			**1**
**h102**										1			**1**
**h103**										1			**1**
**h104**										1			**1**
**h105**										1			**1**
**h106**										2		1	**3**
**h107**										1			**1**
**h108**										1			**1**
**h109**										1			**1**

**h110**										1			**1**
**h111**										1			**1**
**h112**										1			**1**
**h113**										1			**1**
**h114**										1			**1**
**h115**										1			**1**
**h116**										1			**1**
**h117**											1		**1**
**h118**											1		**1**
**h119**											1		**1**
**h120**											1		**1**
**h121**											1		**1**
**h122**											2		**2**
**h123**											1		**1**
**h124**											1		**1**
**h125**											1		**1**
**h126**												1	**1**
**h127**												1	**1**
**h128**												1	**1**
**h129**												1	**1**
**h130**												1	**1**
**h131**												1	**1**
**h132**												1	**1**
**h133**												1	**1**
**h134**												1	**1**
**h135**												1	**1**
**h136**												1	**1**
**h137**												1	**1**
	**23**	**18**	**10**	**10**	**10**	**10**	**10**	**16**	**20**	**20**	**11**	**16**	**174**

Supplementary Table S3. Tamura-Nei genetic distance matrix of 12 MG populations: between (below) and within populations (in diagonal).

	INT	LTS	LOG	ERD	MGG	MZG	NOM	BOG	MAN	BGN	ALT	Ref.
**INT**	**0.034**											
**LTS**	0.029	**0.020**										
**LOG**	0.035	0.032	**0.036**									
**ERD**	0.031	0.023	0.034	**0.022**								
**MGG**	0.034	0.027	0.037	0.029	**0.034**							
**MZG**	0.029	0.021	0.031	0.023	0.027	**0.022**						
**NOM**	0.034	0.028	0.037	0.029	0.033	0.028	**0.035**					
**BOG**	0.028	0.021	0.030	0.024	0.027	0.021	0.028	**0.021**				
**MAN**	0.033	0.027	0.035	0.029	0.033	0.027	0.033	0.027	**0.033**			
**BGN**	0.029	0.021	0.032	0.022	0.027	0.021	0.027	0.021	0.027	**0.021**		
**ALT**	0.036	0.032	0.039	0.032	0.037	0.032	0.036	0.032	0.036	0.031	**0.040**	
**Ref.**	0.036	0.030	0.039	0.032	0.035	0.030	0.035	0.030	0.035	0.030	0.039	**0.039**

Supplementary Table S4. Shared haplotypes among Mongolian goats and those from other countries

Haplotypes	Shared animals	No. of animals	Populations
hap-2	mng02, 08, 116	3	2IKN, 1MAN
hap-12	mng13, ref-1	2	1IKN, 1Ref.
hap-14	mng15, 18	2	2IKN
hap-17	mng19, 137	2	1IKN, 1BGN
hap-19	mng21, 103, 122, 123	4	1IKN, 1BOG, 2MAN
hap-22	mng24, 25	2	2LTS
hap-23	mng26, 31	2	2LTS
hap-27	mng30, 36, 40	3	2LTS
hap-29	mng33, 35	2	2LTS
hap-34	mng41, 87	2	1LOG, 1NOM
hap-35	mng42, 106	2	1LOG, 1NOG
hap-36	mng43, 49	2	2LOG
hap-38	mng45, ref-2	2	1LOG, 1Ref.
hap-45	mng53, 54, 150	3	2ERD, 1ALT
hap-47	mng56, 60	2	2ERD
hap-49	mng58, 59	2	2ERD
hap-52	mgg63, mng147	2	1MGG, 1BGN
hap-56	mgg67, ref-3	2	1MGG, 1Ref.
hap-58	mgg69, mng86	2	1MGG, 1NOM
**hap-60**	**mng71, mzg79, mng90, 91 hua, lei, SNW, SNW**	**8**	**INT, MZG, NOM, HUA, LEI, SNW**
hap-66	mzg77, mng92, 100	3	1MZG, 2BOG
hap-74	mng88, 89	2	2NOM
hap-75	mng93, 119	2	1BOG, 1MAN
hap-78	mng96, 107	2	1BOG, 1MAN
**hap-88**	**mng110, hua, hua, KNG**	**4**	**MAN, HUA, KNG**
hap-91	mng114, 115	2	2MAN
hap-106	mng134, 144, ref-4	3	2BGN, 1Ref.
**hap-118**	**mng149, hua**	**2**	**1ALT, 1HUA**
hap-122	mng154, 155	2	1ALT

mng## = individual ID of Mongolian goats in this study

Supplementary Table S5. The pairwise *Fst* distance matrix among a total of 36 breeds

	1	2	3	4	5	6	7	8	9	10	11	12	13	14	15	16	17	18	19	20	21	22	23	24	25	26	27	28	29	30	31	32	33	34	35	36
1 INT	0.0																																			
2 LTS	0.04	0.00																																		
3 LOG	0.02	0.14	0.00																																	
4 ERD	0.06	0.08	0.15	0.00																																
5 MGG	-0.01	0.00	0.07	0.04	0.00																															
6 MZG	0.01	-0.02	0.09	0.05	-0.04	0.00																														
7 NOM	-0.03	0.00	0.04	0.02	-0.06	-0.03	0.00																													
8 BOG	0.02	0.03	0.06	0.09	-0.02	-0.02	0.00	0.00																												
9 MAN	-0.02	0.02	0.01	0.03	-0.02	-0.01	-0.04	0.00	0.00																											
10 BGN	0.04	0.01	0.15	0.01	0.00	-0.02	-0.01	0.03	0.01	0.00																										
11 ALT	-0.02	0.07	0.01	0.01	0.00	0.03	-0.04	0.06	-0.01	0.05	0.00																									
12 Ref.	-0.02	0.03	0.04	0.02	-0.04	-0.01	-0.05	0.01	-0.02	0.01	-0.02	0.00																								
13 GO	0.18	0.28	0.20	0.28	0.18	0.24	0.19	0.20	0.21	0.28	0.20	0.16	0.00																							
14 KG	0.10	0.17	0.14	0.18	0.09	0.13	0.09	0.09	0.12	0.17	0.13	0.09	0.04	0.00																						
15 TG	0.05	0.06	0.14	0.13	0.04	0.05	0.05	0.04	0.04	0.06	0.08	0.05	0.24	0.12	0.00																					
16 BA	0.08	0.09	0.16	0.12	0.03	0.06	0.07	0.03	0.07	0.08	0.11	0.08	0.22	0.11	0.05	0.00																				
17 BAT	0.11	0.21	0.11	0.21	0.12	0.18	0.13	0.14	0.13	0.22	0.12	0.11	0.03	0.05	0.15	0.15	0.00																			
18 BE	0.10	0.12	0.18	0.15	0.06	0.11	0.09	0.07	0.10	0.13	0.12	0.09	0.25	0.12	0.11	0.06	0.19	0.00																		
19 AR	0.09	0.15	0.17	0.18	0.06	0.11	0.08	0.06	0.10	0.14	0.12	0.08	0.20	0.09	0.07	0.04	0.16	0.12	0.00																	
20 CHE	0.08	0.15	0.13	0.13	0.08	0.09	0.04	0.12	0.09	0.12	0.06	0.04	0.15	0.12	0.16	0.17	0.14	0.19	0.15	0.00																
21 GUB	0.04	0.12	0.10	0.12	0.07	0.08	0.02	0.09	0.06	0.11	0.02	0.02	0.15	0.10	0.11	0.15	0.10	0.18	0.14	-0.05	0.00															
22NUA	0.09	0.09	0.19	0.04	0.06	0.07	0.05	0.13	0.04	0.05	0.08	0.04	0.33	0.24	0.16	0.18	0.29	0.23	0.23	0.18	0.20	0.00														
23LEI	0.22	0.33	0.27	0.22	0.22	0.27	0.18	0.31	0.23	0.27	0.18	0.16	0.28	0.28	0.34	0.35	0.29	0.37	0.33	0.05	0.12	0.28	0.00													
24SNW	0.14	0.20	0.22	0.10	0.12	0.15	0.09	0.21	0.14	0.13	0.10	0.08	0.27	0.23	0.24	0.25	0.25	0.26	0.25	0.03	0.09	0.14	0.01	0.00												
25KNG	0.14	0.21	0.24	0.05	0.15	0.19	0.10	0.26	0.10	0.12	0.09	0.07	0.38	0.30	0.30	0.33	0.34	0.38	0.36	0.17	0.20	0.03	0.20	0.08	0.00											
26 MJ	0.16	0.21	0.29	0.28	0.16	0.23	0.17	0.18	0.17	0.22	0.24	0.16	0.26	0.18	0.20	0.19	0.23	0.27	0.22	0.26	0.25	0.30	0.42	0.32	0.45	0.00										
27 dDS	0.21	0.34	0.31	0.37	0.27	0.36	0.26	0.31	0.22	0.33	0.26	0.19	0.36	0.30	0.25	0.37	0.31	0.48	0.35	0.31	0.28	0.42	0.42	0.37	0.52	0.43	0.00									
28GER	0.17	0.27	0.29	0.32	0.21	0.28	0.22	0.23	0.18	0.26	0.22	0.15	0.32	0.24	0.14	0.28	0.27	0.37	0.27	0.28	0.24	0.36	0.43	0.36	0.48	0.40	0.10	0.00								
29 ML	0.16	0.29	0.28	0.34	0.23	0.31	0.21	0.26	0.17	0.28	0.22	0.14	0.32	0.25	0.17	0.32	0.29	0.42	0.30	0.27	0.24	0.37	0.42	0.36	0.51	0.38	0.10	0.05	0.00							
30 PiN	0.14	0.20	0.25	0.30	0.15	0.23	0.16	0.19	0.14	0.23	0.20	0.13	0.24	0.18	0.21	0.21	0.20	0.29	0.26	0.25	0.25	0.32	0.43	0.33	0.50	0.02	0.48	0.44	0.45	0.00						
31 IG	0.03	0.08	0.07	0.07	-0.03	0.02	-0.02	0.04	0.02	0.05	0.01	-0.02	0.14	0.09	0.07	0.09	0.10	0.13	0.08	0.01	0.01	0.11	0.11	0.06	0.14	0.18	0.22	0.19	0.20	0.19	0.00					
32 ING	0.53	0.68	0.57	0.71	0.61	0.69	0.61	0.65	0.56	0.68	0.56	0.52	0.31	0.48	0.69	0.69	0.44	0.73	0.68	0.50	0.54	0.73	0.56	0.60	0.78	0.70	0.74	0.73	0.76	0.72	0.51	0.00				
33 LOG	0.32	0.45	0.35	0.37	0.33	0.39	0.29	0.42	0.34	0.41	0.28	0.26	0.33	0.36	0.47	0.46	0.36	0.49	0.42	0.10	0.19	0.43	-0.02	0.11	0.37	0.53	0.52	0.54	0.54	0.53	0.19	0.58	0.00			
34 PG	0.09	0.20	0.08	0.14	0.08	0.14	0.05	0.15	0.10	0.18	0.05	0.06	0.14	0.15	0.20	0.19	0.12	0.21	0.17	0.03	0.05	0.18	0.04	0.08	0.15	0.27	0.25	0.27	0.25	0.24	0.01	0.37	0.08	0.00		
35 Kag	-0.03	-0.01	0.07	0.03	-0.05	-0.03	-0.05	0.00	-0.03	-0.01	-0.02	-0.05	0.18	0.05	-0.05	0.03	0.11	0.06	0.05	0.06	0.04	0.07	0.25	0.13	0.20	0.17	0.26	0.15	0.18	0.18	0.00	0.69	0.37	0.10	0.00	
36 Kyg	0.04	0.19	-0.02	0.19	0.10	0.16	0.09	0.13	0.05	0.20	0.05	0.09	0.20	0.17	0.19	0.19	0.11	0.28	0.21	0.14	0.10	0.24	0.26	0.22	0.28	0.32	0.31	0.31	0.32	0.29	0.08	0.56	0.33	0.06	0.13	0.0

Highlighted area indicated *Fst* values among MG populations

Highlighted area indicated *Fst* values among MG populations and foreign breeds

Highlighted area indicated no important *Fst* values among foreign breeds
